# Wild Fruits of *Amelanchier ovalis* Medik. (Rosaceae): A Valuable Neglected Resource of Nutrients and Bioactive Compounds

**DOI:** 10.1007/s11130-026-01497-x

**Published:** 2026-04-10

**Authors:** Patricia García-Herrera, Erika N. Vega, José Ignacio Alonso-Esteban, Javier Tardío, Adriana K. Molina, Filipa Mandim, Tânia C. S. P. Pires, Lillian Barros, María Luisa Pérez-Rodríguez, Montaña Cámara, Virginia Fernández-Ruiz, Patricia Morales

**Affiliations:** 1 Dpto. Nutrición y Ciencia de los Alimentos, Facultad de Farmacia, Universidad Complutense de Madrid (UCM), Plaza Ramón y Cajal s/n, Madrid, 28040 Spain; 2 Instituto Madrileño de Investigación y Desarrollo Rural, Agrario y Alimentario (IMIDRA), Finca El Encín. Apdo. 127, Alcalá de Henares, Madrid, 28800 Spain; 3 CIMO, LA SusTEC, Instituto Politécnico de Bragança, Campus de Santa Apolónia, Bragança, 5300-253 Portugal

**Keywords:** Wild edible plants, Nutritional composition, Bioactive compounds, Biological activities, Preservative properties

## Abstract

*Amelanchier ovalis* Medik., is a neglected wild edible fruit traditionally consumed in several European regions and adapted to diverse agroecological conditions. The present study aimed to provides a preliminary physico-chemical and characterization of the fruits collected in central Spain. The analysis focused on fatty acids, tocopherols, soluble sugars, organic acids, and phenolic compounds, alongside an evaluation of the antioxidant and antimicrobial capacity. The results indicate that this wild edible fruit is characterized by a high proportion of unsaturated fatty acids, particularly oleic (34%) and linoleic (30%) acids, and contain appreciable amounts of tocopherols (28 mg/100 g, fw). The presence of sorbitol stands out in this species as a soluble sugar (> 2 g/100 g, fw), and the organic acid profile revealed a balanced composition. A high total phenolic content was observed, characterized by hydroxybenzoic and hydroxycinnamic acids. In vitro assays demonstrate radical scavenging capacity and inhibitory activity against selected pathogenic bacterial and fungal strains. Furthermore, no in vitro cytotoxicity was evidenced in non-tumour hepatic cell lines (PLP2). These findings provide a baseline characterization of the nutritional and bioactive compounds profile of *A. ovalis* fruits, suggesting their revalorization for its consumption as part of the Mediterranean diet and as a potential technological functional ingredient, although further studies on bioavailability and in vivo efficacy are required.

**Supplementary Information:**

The online version contains supplementary material available at 10.1007/s11130-026-01497-x.

## Introduction

The search for sustainable and healthy food sources has increased due to the current global environmental challenges. Globalization and commercial trade have contribute to higher pollution levels, and changes in food production are needed to decrease greenhouse gas emissions. Meeting the Sustainable Development Goals (SDG) proposed in the 2030 Agenda is a significant challenge for both industry and governments around the world. The last trends to achieve these goals included the revalorization of wild edible fruits, which have historically served as traditional food sources across different cultures. These fruits represent a viable source of nutrients and have historically supported the survival of different populations during periods of wars or famine [1, 2]. Due to their nutritional value, confirmed by some authors [3, 4], they can be contemplated as sustainable resources to consider in a healthy diet.

**Fig. 1:**
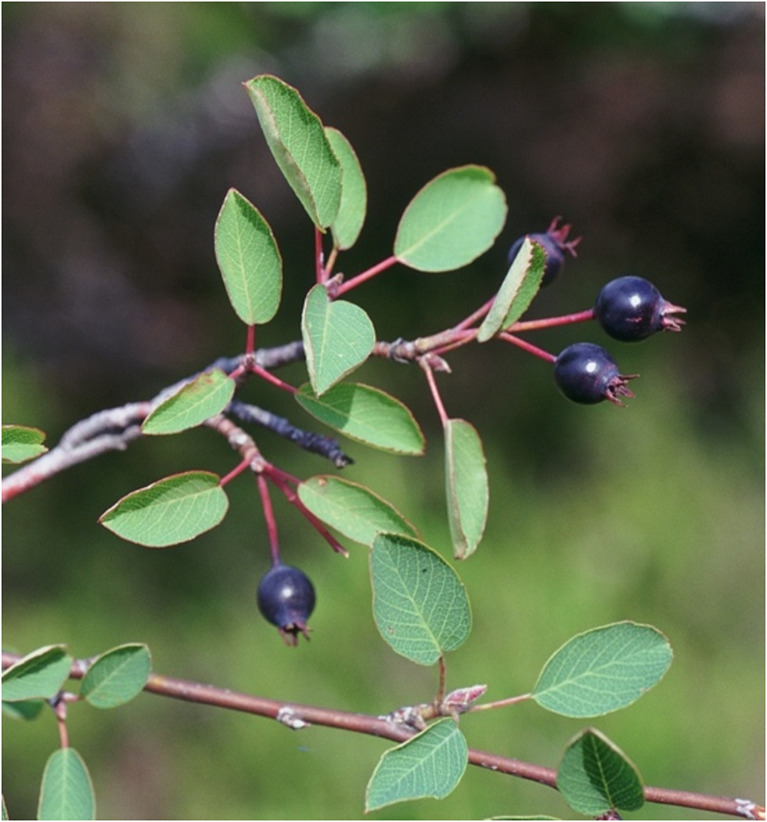
Branch of *Amelanchier ovalis* Medik. with some ripe fruits. (source: Javier Tardio)

In the Mediterranean region, many wild plants with edible fruits belong to the Rosaceae family [5, 6]. One of the genera of this family is *Amelanchier*, that includes 24 accepted species, primarily distributed in North American with a few species in Europe and Eastern Asia [7]. *Amelanchier ovalis* Medik., commonly known in English as “snowy mespilus”, or “serviceberry” [8], is broadly distributed across in Central and Southern Europe, Asia Minor, and along the North coast of Africa [7]. It is a deciduous shrub reaching up to 3 m in height, with upright occasionally arched branches, and petiolate, elliptical leaves, blade up to 4 × 3 cm and as shown in Fig. 1, the fruits are blackish globose pomes, approximately 1 cm in diameter [9]. In Spain, *A. ovalis* inhabits open forests, forest edges, and sparse scrublands, mainly in rocky areas of mountainous regions between 300 and 2,500 m of altitude. It is especially common in the mountain systems of the eastern half of the country, where it occupies limestone terrains, although it also occurs in some mountainous areas of the centre and northwest that are developed on siliceous rocks [9].

Similar to other American species of this genus [10, 11], the edible fruits of *A. ovalis* have been traditionally consumed in several European countries [5, 8]. Although these fruits are not among the most commonly consumed wild berries in Spain, likely due to the limited abundance of the species, they have traditionally been gathered and eaten in various regions, generally consumed raw, directly in the field, and occasionally served as a dessert [5, 12]. Ethnobotanical reports suggest that the fruits were historically regarded as restorative, or used to alleviate headaches. Medicinal uses of other plant parts have also been documented, including their application as antihypertensive agents and in the treatment of bronchial ailments, typically through decoctions or infusions prepared from stems with leaves and flowers, or exclusively from young stems with leaves, respectively [12]. The flowers have likewise been incorporated into certain herbal liqueurs. Similarly, the fruits *Amelanchier alnifolia* Nutt. were historically used both raw and processed and even used by European explorers to prevent nutritional deficiencies, such as scurvy, highlighting the functional potential of the genus [10].

The ecological characteristics and its cultural use history of *A. ovalis* suggest that this fruit is a promising candidate to be included in a sustainable diet. The consumption and revalorization of this fruit aligns with several SDGs, specifically with goals 2, 3, 8, 11, 12 and 15. Its revaluation and consumption could improve nutritional diversity, promote local and sustainable agriculture, encourage the creation of rural jobs, and the protection of regional biodiversity.

Despite its potential, information about its composition as well as its bioactivity is scarce [13–15]; notably, there are few scientific references about aspects such as antioxidant and antifungal activities. In view of the interest in using local resources, the main objective of this research is the revalorization of these wild edible fruits gathered in Spain in order to promote its consumption as part of the Mediterranean diet, as well as to highlight its promising use as a food ingredient due to its functional properties (preservative potential). To achieve this objective, two specific objectives were proposed: (1) To characterize some nutrients and bioactive compounds in the edible fruit portion of *A. ovalis* (in terms of fatty acids, tocopherols, soluble sugars, organic acids and phenolic compounds), and (2) to study the bioactivity of *A. ovalis* fruit (in terms of antioxidant properties, antibacterial and antifungal activity).

## Materials and Methods

### Samples

To account for some of the potential environmentally driven variability, the fruits were collected from two wild populations of *Amelanchier ovalis* located at two different sites of Central Spain, with different soils composition and at a slight different altitude (see Table 1). The samples were harvested at the optimal maturing state, in July 2022, when the fruits were completely ripened, in accordance with permits Ref. PN-NC_022022 and Ref. ABSCH-IRCC-ES-262067-1 issued by the Spanish Ministry of Agriculture, Fisheries, and Food (MAPA). Samples from both origins were pooled and processed as a single batch. A portion of the fresh fruit mixture was reserved for immediate analysis of moisture, °Brix, pH, and titratable acidity. The remaining fruit was frozen and subsequent freeze-drying at − 80 °C and 0.029 mbar (Freezone; 4.5 L; LABCONCO, Fort Scott, KS, USA). Seeds were then removed to obtain a homogeneous analytical matrix corresponding to the dry edible portion of the fruit, including both peel and fleshy tissues. The freeze-dried samples were stored at −20 °C (in dark and dry conditions) for subsequent analysis. Results obtained from freeze-dried samples were converted to fresh weight (fw) basis, considering the initial moisture content of the fruit samples, using the following formula: Value_fw_ = Value_dw_ X ((100 - % moisture)/100).

**Table 1:** Collection sites, coordinates and ecological conditions were *Amelanchier ovalis* fruits were sampled

Municipality (province)	Geographical coordinates	Ecological conditions
Valdemeca (Cuenca)	Latitude: 40° 14’ 47.9” N; Length: 1° 45’ 39,6” W	Limestone soils, 1300 m
Bustarviejo (Madrid)	Latitude: 40° 50’ 54.5” N Length: 3° 41’ 38.9” W	Siliceous soils, 1100 m

## Methods

Detailed procedures for physico-chemical, chemical, bioactivity determinations and statistical analysis used are provided in the supplementary materials.

## Results and Discussion

There is scarce information about genus *Amelanchier* and even less about *A. ovalis.* Thus, the comparison of this species will often be performed with other red wild fruits, most of them of the Maleae tribe.

The physico-chemical characteristics of *A. ovalis* are summarized in Table 2. Sagandyk et al. [13] reported a moisture of 51.13 g/100 g (fw), which is lower than data the value obtained in this study. Environmental conditions could be related with this difference. Furthermore, the sample in the present study were analysed after seeds removal, as seeds typically possess the lowest moisture content, their exclusion likely contributed to the higher moisture levels observed. Conversely, other authors reported higher moisture content (84.63 g/100 g) for other *Amelanchier* spp [16].

**Table 2:** Physico-chemical determinations (fw) in edible fruit portion of *Amelanchier ovalis*

Physico-chemical parameters	
Moisture (g/100 g)	65.28 ± 1.99
pH	5.37 ± 0.03
TA (mL NaOH/100 g)	254.07 ± 23.03
°Brix	0.80 ± 0.00

Data are expressed as mean ± standard deviation (*n* = 3). TA: Titratable acidity

Regarding acidity, the pH of A. ovalis was notably higher than the range of 4.06–4.61 reported by Tamayo-Vives et al. [17] for other Maleae tribe fruits, such as *Crataegus monogyna* Jacq. and *Sorbus aria* (L.) Crantz. Vega et al. [3] studied other wild red fruits like *Fragaria vesca* L., *Prunus avium* L. and *Vaccinium myrtillus* L. In this case, *A. ovalis* presented higher pH values (5.37) than these fruits (3.35–4.01). However, the titratable acidity (TA) in this study was higher, than the average value reported by the same author (190.60 mL NaOH/100 g, fw). Suggesting a high concentration of organic acids despite the higher pH.

Data about fatty acids’ composition of *A. ovalis* fruit edible portion remain limited. The analysed fruit presented 3.30 g/100 g (fw) of crude fat content and, as shown in Table 3, the profile is characterized by a high proportion of unsaturated fatty acids content, particularly oleic and linoleic acid (34.18 and 29.97%, respectively), followed by palmitic acid (16.46%) and in minor content α-linolenic acid (5.15%). Other authors, such as Sagandyk et al. [13] studied the fatty acids profile of the same species and they found linoleic acid as major fatty acid (44%) in the same species, followed by oleic acid (21%). These differences could be due to the exclusion of seeds in this study, which typically alter the lipid profile in fruits.

**Table 3:** Lipophilic compounds analysis in *Amelanchier ovalis* edible fruit portion

Lipophilic compounds		
Fatty acids (relative percentage, %)		
C8:0	0.140 ± 0.008	
C10:0	0.266 ± 0.018	
C12:0	0.717 ± 0.040	
C14:0	1.366 ± 0.085	
C15:0	0.559 ± 0.033	
C16:0	16.46 ± 0.55	
C16:1	0.667 ± 0.052	
C17:0	0.464 ± 0.016	
C18:0	4.488 ± 0.036	
C18:1*n*9	34.175 ± 0.075	
C18:2*n*6	29.97 ± 0.56	
C18:3*n*3	5.15 ± 0.098	
C20:0	1.35 ± 0.035	
C20:1	0.340 ± 0.028	
C21:0	0.291 ± 0.014	
C20:3n3	0.621 ± 0.034	
C22:0	1.499 ± 0.004	
C23:0	0.271 ± 0.017	
C24:0	1.206 ± 0.122	
**Tocopherols (mg/100 g**,** fw)**		
α-tocopherol	27.268 ± 0.351	
β-tocopherol	1.204 ± 0.051	
Total tocopherols	28.472 ± 0.300	

Data are expressed as mean ± standard deviation (n=3)

Comparing to other wild red fruits, such as *Arbutus unedo* L., *Crataegus monogyna* Jacq., *Prunus spinosa* L. and *Rubus ulmifolius* Schott. or *Myrtus communis* L [18]. the main fatty acids were consistently linoleic, oleic and palmitic acid, although with higher values of linoleic acid and lower of oleic acid (69.17 and 9.21%, respectively), according to Sagandik et al. [13]. Similar data were obtained by Ilhan et al. [19] with linoleic (35.91%), palmitic (19.19%) and oleic (11.8%) acids as the predominant in Sea buckthorn (*Hippophae rhamnoides* subsp. *caucasica* Rousi).

Regarding the tocopherol content, most of the published studies found were carried out in seed oils. There are scarce references about *Amelanchier* fruits and none of them from *A. ovalis*. The berries of *Amelanchier alnifolia* Nutt. have been reported to contain between 2.16 and 2.71 mg/Kg dw of total tocopherols [20], significantly lower than results obtained in this study (9885.4 mg/kg dw).

Regarding soluble sugars analysis (Table 4), the literature related to *A. ovalis* remains limited. Sagandyk et al. [13] reported glucose and fructose as main soluble sugars in *A. ovalis* (4.6 and 3.5 g/100 g, respectively), which aligns closely with the results obtained in our study. Other *Amelanchier* species, such as *A. lamarckii* F.G. Schroed. presented the same sugar profile than *A. ovalis* in the present study, where the main soluble sugars were glucose, fructose and sorbitol. However, sugar contents for *A. lamarckii* fruit reported by Mikulic-Petkovsek et al. [21] were considerably lower compared to *A. ovalis* (*A. lamarckii*: 610 mg of glucose/100 g fw; 640 mg of fructose/100 g fw and 510 mg of sorbitol/100 g). Additionally, other wild red fruits presented higher soluble sugars content, as in the case of *Rosa canina* L. (7248, 8363 and 455 mg/100 g (fw) of glucose, fructose and saccharose, respectively) [22]. The same authors found a lower glucose content in *Fragaria vesca* L. (2527 mg/100 g, fw) but higher fructose and saccharose amount (6079 and 560 mg/100 g fw, respectively). Ruiz-Rodriguez et al. [23] studied other related Spanish wild edible fruit (*Arbutus unedo* L.), and reported higher fructose and glucose content, but similar saccharose content (9650, 4660 and 130 mg/100 g, fw) comparing with *A. ovalis* results.

**Table 4:** Hydrophilic compounds analysis (fw) in *Amelanchier ovalis* edible fruit portion

Hydrophilic compounds	
Soluble sugars (g/100 g, fw)	
Fructose	3.374 ± 0.072
Sorbitol	2.274 ± 0.059
Glucose	3.365 ± 0.264
Saccharose	0.127 ± 0.003
Total soluble sugar (g/100 g, fw)	9.140 ± 0.392
**Organic acids (mg/100 g**,** fw)**	
Oxalic acid	377.4 ± 2.2
Quinic acid	679.6 ± 24.2
Malic acid	488.4 ± 6.2
Shikimic acid	29.2 ± 0.8
Citric acid	219.5 ± 8.8
Total Organic acids (mg/100 g, fw)	1794.0 ± 42.3
**Total Phenolic compounds**	
TPC-FB (mg GAE/100 g, fw)	2065.4 ± 94.2
HBA (mg GAE/100 g, fw)	584.6 ± 42.8
HCA (mg FAE/100 g, fw)	520.7 ± 32.1
FLAV (mg QE/100 g, fw)	384.7 ± 17.2
TAC (mg cya-3-glu/100 g, fw)	103.6 ± 5.7

Data are expressed as mean ± standard deviation (n = 3). TPC-FB: Total polyphenols content-Fast Blue, HBA: total hydroxybenzoic acid, HCA: total hydroxycinnamic acid. Flav: total flavonols. TAC: Total anthocyanin content, cya-3-glu: cyanidin-3-O-glucoside; GAE: gallic acid equivalent; FAE: ferulic acid equivalent; QE: quercetin equivalent; fw: fresh weight

The content of sorbitol stands out in *A. ovalis*, as this sugar is generally absent in many common wild red fruits. Mikulic-Petkovsek et al. [11] studied the composition of 25 different wild red fruits and found that only three species from the Maleae tribe (*Aronia melanocarpa* (Michx.) Elliott, *Sorbus aucuparia* L. and *Amelanchier canadensis* (L.) Medik.) presented sorbitol, with higher contents than those reported in this study (4620, 13410 and 5330 mg/100 g fw, respectively).

**Fig. 2:**
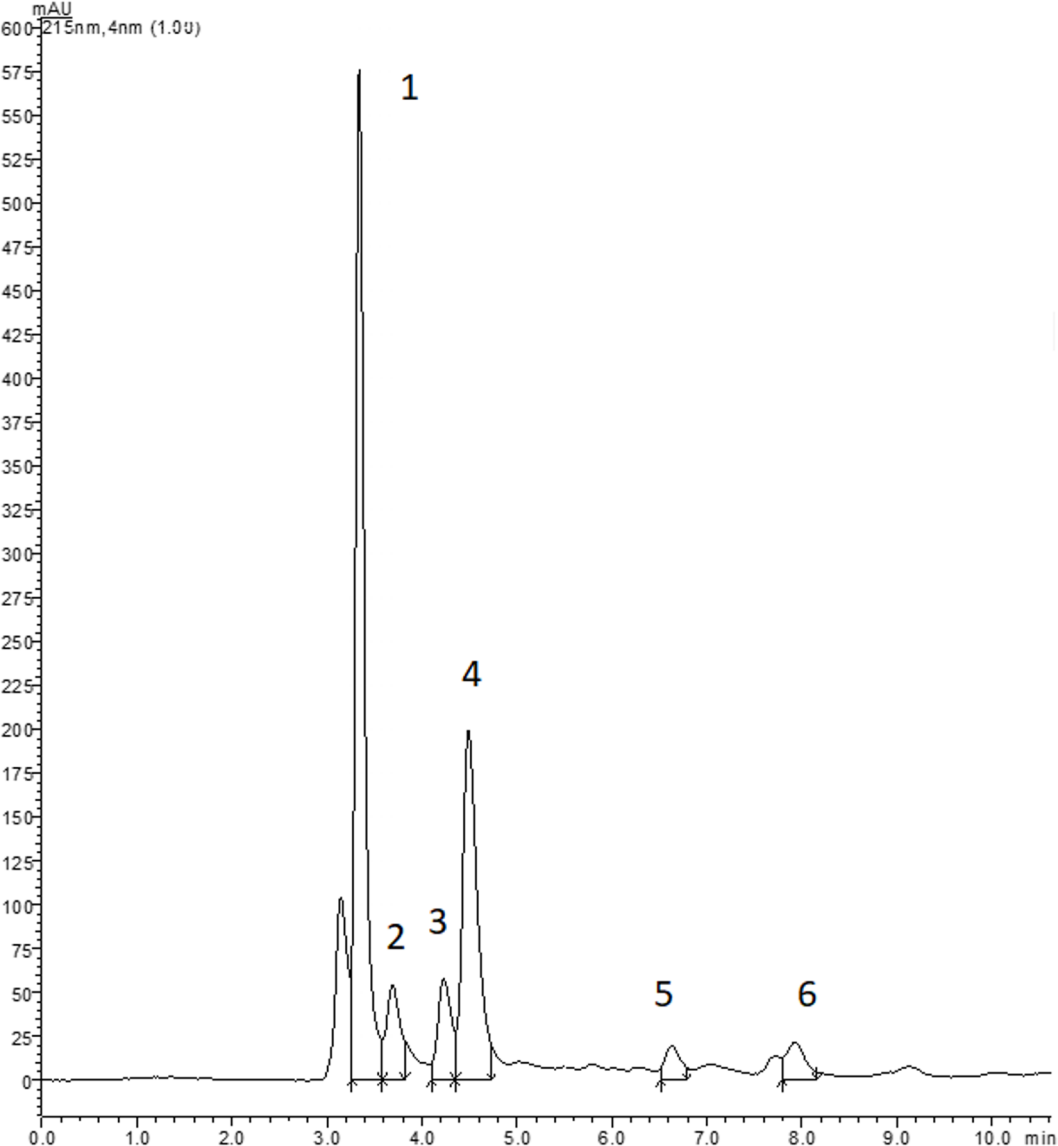
Organic acids chromatogram of *A. ovalis fruits.* 1: oxalic acid; 2: quinic acid; 3: malic acid; 4: shikimic acid; 5: citric acid; 6: fumaric acid

The organic acids profile of A. ovalis is illustrated in Fig. 2, with corresponding data included in Table 4. Quinic acid was the major organic acid found in the analysed fruits, with a content of 679.6 g/100 g (fw), followed by malic and oxalic acids, both with a content lower than 500 mg/100 g (fw). Sagandyk et al. [13] studied *A. ovalis* from Kazakhstan, and reported the same primary organic acids; however, malic acid was reported as the most abundant constituent (3.5 g/100 g, dw). Considering the moisture content of *A. ovalis*, the content of malic acid concentration in our study was 1.4 g/100 g (dw), which is notably lower that the value previously reported by the authors. Despite these differences, citric and shikimic acids were found in minor proportion in both studies, and the total organic acid content remained similar. Specifically, Sagandyk et al. [13] reported a total organic acids content of 4.7 g/100 g (dw), while the value found in our study was 1.794 g/100 g fw (equally to 5.1 g/100 g, dw).

*A. ovalis* is an interesting fruit due to its high phenolic content. As shown in Table 4, *A*. *ovalis* fruits presented a total phenolic content (TPC) of 2,065.4 mg GAE/100 g, fw (equally to 59.5 mg/g dw), which is substantially higher than the value reported by Ochmian et al. [16] for the same fruit (356 mg GAE/100 g, fw). Moreover, *A. ovalis* presented higher phenolic content than other species of the same genus, such as *A. alnifolia* and *Amelanchier spicata* (Lam.) K. Koch which presented TPC values of 261 and 219 mg GAE/100 g (fw), respectively [16].

Additionally, *A. ovalis* also presented higher TPC value compared to other wild fruits of the same taxonomic tribe from the Iberian Peninsula, such as *C. monogyna* and *S. aria* (1,702 to 6637 mg GAE/100 g dw, respectively) [17], being *A. ovalis* in the upper end of the range (5,948 mg GAE/100 g, dw). Other wild fruits of the Rosaceae family such as *Prunus avium* (362 mg GAE/100 g, fw) and *Fragaria vesca* exhibited lower TPC content (362 and 1315 mg GAE/100 g, fw, respectively) [3].

Regarding phenolic families, *A. ovalis* showed high concentrations of hydroxybenzoic acids (HBA), as well as significant levels of hydroxycinnamic acids (HCA), the latter of which have been reported as the most abundant phenolic compounds in other *Amelanchier* species [24]. Additionally, it presented significant amounts of total anthocyanins (TAC), which likely contributed to its characteristic purple colour. Moreover, the values of phenolic families found in this study (1,709.4 mg GAE/100 g (dw), 1,522.5 mg FAE/100 g (dw), 1,124.9 mg QE/100 g (dw) and 302.9 mg cyd-3-glu/100 g (dw), respectively) were remarkedly higher than those previously reported for this fruit by Mikulic-Petkovsek et al. [21] with values of HBA of 0.081 mg GAE/100 g (dw), HCA of 0.52 mg FAE/100 g (dw), F of 82.9 mg QE/100 g (dw) and TAC of 85.3 mg cyd-3-glu/100 g (dw).

Total hidroxibenzoic acids (HBA), total flavonols (FLAV) and total anthocyanins content (TAC) of *A. ovalis* edible fruit portion was within the ranges reported by Tamayo-Vives et al. [17] for *C. monogyna* and *S. aria*. However, total hydroxycinnamic acid content (HCA) was notably higher compared with the results obtained in the present study (1,501 vs. 841 mg FAE/100 g, dw). Other wild fruits of interest that belongs to the same family, such as *Rubus plicatus* L., generally show a greater amount of HCA over HBA [15], while fruits such as, *Rubus ulmifolius* Schott. present remarkable higher contents of total anthocyanins, reaching levels almost as high as their content of phenolic acids [25]. These comparisons highlight the distinctiveness of the phenolic composition of *A. ovalis* being mostly an exceptional source of phenolic acids among wild edible fruits.

Thanks to the richness of *A. ovalis* in bioactive compounds, a strong antioxidant activity was evidenced in all three in vitro assays (Table 5). These results are qualitatively consistent with the high antioxidant potential previously reported for this fruit, for example, a 93.1% of radical scavenging activity through DPPH was evidenced in fruits cultivated in Greece [8] and an EC_50_ of 0.42 mg/mL was measured by ABTS in an extract from Siberian fruits [26]. While these metrics (TE values, inhibition percentages or EC_50_) impede a direct quantitative comparison, they collectively underscore the significant radical scavenging properties of *A. ovalis*.

**Table 5:** Biological activities of *Amelanchier ovalis* edible fruit portion

ANTIOXIDANT ACTIVITY												
Antioxidant activity in vitro chemical												
Q-Folin-Ciocâlteu (mg GAE/100 g, fw)	911.4 ± 64.7											
Q-DPPH (mg TE/100 g, fw)	414.1 ± 19.7											
Q-FRAP (mg TE/100 g, fw)	3140.8 ± 96.1											
**Antioxidant activity** **in vitro** **biological**												
	60 min		120 min									
OxHLIA (IC_50_, µg/mL)	107.9 ± 4.7		190.5 ± 6.1									
**ANTIBACTERIAL ACTIVITY**												
	**AO(1 mg/mL)**		**Streptomicin(1 mg/mL)**		**Methicilin(1 mg/mL)**			**Ampicillin(10 mg/mL)**				
**Gram-negative bacteria**	**MIC**	**MBC**	**MIC**	**MBC**	**MIC**	**MBC**		**MIC**		**MBC**		
*Enterobacter cloacae*	> 10	> 10	0.007	0.007	n.t.	n.t		0.15		0.15		
*Escherichia coli*	10	> 10	0.01	0.01	n.t.	n.t.		0.15		0.15		
*Pseudomonas aeruginosa*	> 10	> 10	0.06	0.06	n.t.	n.t.		0.63		0.63		
*Salmonella* Enteriditis	5	> 10	0.007	0.007	n.t.	n.t.		0.15		0.15		
*Yersinia enterocolitica*	> 10	> 10	0.007	0.007	n.t.	n.t.		0.15		0.15		
**Gram-positive bacteria**												
*Bacillus cereus*	> 10	> 10	0.007	0.007	n.t.	n.t.		n.t.		n.t.		
*Listeria monocytogenes*	> 10	> 10	0.007	0.007	n.t.	n.t.		0.15		0.15		
*Staphylococcus aureus*	> 10	> 10	0.007	0.007	0.007	0.007		0.15		0.15		
**ANTIFUNGAL ACTIVITY (hydroethanolic extract)**												
	**AO** **(10 mg/mL)**		**Ketoconazole** **(1 mg/mL)**									
	**MIC**	**MFC**	**MIC**	**MFC**								
*Aspergillus brasiliensis*	1.25	> 10	0.06	0.125								
*Aspergillus fumigatus*	> 10	> 10	0.5	1.0								

Data are expressed as mean ± standard deviation (n=3). GAE: gallic acid equivalent; TE: trolox equivalent; IC50: extract concentration providing 50 % of antioxidant activity. MIC: minimum inhibitory concentration; MBC: minimum bactericidal concentration; MFC: minimum fungicidal concentration; n.t.: not tested. IC 50 for Trolox OxHLIA assay = 21.8 ± 0.30 μg/mL

Moreover, the antioxidant capacity of *A. ovalis* (expressed in dry weigh basis: 9,047.4 mg TE/100 g, by FRAP; 1,192.9 mg TE/100 g, by DPPH and 2,625.4 mg GAE/100 g, by Folin Ciocâlteu) exceeded values previously reported for other *Amelanchier* species, including *A. alnifolia.* For which were reported values of 4,480 mg TE/100 g (dw), by FRAP and between 2417.80 and 6,342.35 mg TE/100 g (dw) by DPPH [20, 27]. Beyond the genus, the antioxidant capacity of *A. ovalis* was also comparable or superior to other fruits within the subfamily Maloideae, such as *Sorbus aria* L. with values of 873.4 mg TE/100 g (dw), through DPPH; 6,085.7 mg TE/100 g (dw), through FRAP and 702 mg GAE/100 g (dw), through Folin-Ciocâlteu [17, 28] and *Crataegus monogyna* Jacq. with reported values of 874.2 mg TE/100 g (dw), through DPPH and 7,434 mg TE/100 g (dw), through FRAP [17, 29]. These findings highlight the potential of *A. ovalis* as a valuable source of bioactive compounds with notable functional properties.

The high antioxidant activity evidenced in many small fruits has been strongly correlated with their high phenolic content [30]. In the genus *Amelanchier*, this correlation is principally attributed to the presence of phenolic acids, anthocyanins and flavonols, being considered as the primary contributors of radical scavenging capacity [20, 26, 27]. The finding of this study aligns with these reports, since *A. ovalis* presented high concentrations of both phenolic acids and anthocyanins.

Additionally, to better integrate the phytochemical composition of *A. ovalis* with the observed antioxidant capacity, a Spearman’ correlation analysis was performed (Table 6). A perfect positive correlation was observed between hydroxycinnamic acids (HCA) and DPPH radical scavenging activity (*r* = 1.000; *p* < 0.001), suggesting that the high concentration of HCA likely contributes through the resonance-enhancing effects of the propenoic side chain [31]. Moreover, a significant strong correlation of DPPH with total anthocyanin content (TAC) (*r* = 0.827; *p* < 0.001) was observed, aligning with the known ability of the catechol structure in the B-ring of several anthocyanins to form highly stable radical species enhancing the radical scavenging capacity [32]. In contrast, hydroxybenzoic acids (HBA) were perfectly correlated with both FRAP and Folin-Ciocâlteu (*r* = 1.000; *p* < 0.001, for both cases), evidencing that HBA are principal responsible for the reducing power derived from electron transfer.

**Table 6:** Spearman’s correlation coefficients (r) between phenolic profile and the antioxidant capacities of *A. ovalis* fruits

	TPC	HBA	HCA	TAC	FC
Folin	0.767^*^	1.000^**^	0.500	0.529	0.500
DPPH	0.569	0.500	1.000^**^	0.827^**^	1.000^**^
FRAP	0.543	1.000^**^	0.500	0.257	0.500

However, further studies, *i.e*., via HPLC, are required to properly characterize the individual phenolic profile and to establish the specific contribution of each compound to the determined antioxidant activity.

On the other hand, this is the first study to evaluate the in vitro biological antioxidant activity of this fruit using these assays (Table 5). Other authors [33] studied another wild fruit as *Prunus spinosa*, and values of IC_50_ of 296 and 509 µg/mL at 60 and 120 min, respectively, were reported. These values were higher than those obtained in the present study (107.9 and 190.5 µg/mL at 60 and 120 min, respectively), indicating that lower concentration of the extract of *A. ovalis* extract was required to inhibit erythrocyte hemolysis. Therefore, juneberry (*A. lamarckii*) demonstrated stronger antioxidant activity than *P. spinosa*. Additionally, Sagandyk et al. [13] analysed the antioxidant activity in *A. ovalis* by other methods (TBARS assay) and they reported a potent antioxidant activity compared to *Aronia melanocarpa*.

It is important to mention that, although *A. ovalis* fruit presented significant antioxidant potential through both chemical and biological in vitro assays, these results are preliminary and to stablish the actual biological efficacy of this fruit, further research is required to confirm if these observed redox capacities translate into measurable health benefits under physiological conditions.

Antibacterial and antifungal activity were also studied (Table 5). To date, only two studies were performed by other authors about antibacterial activity and only one about antifungal activity [14]. Both studies focused primarily on effects related to human health. In contrast, in this study the antifungal activity was studied as potential natural food preservative, since *Aspergillus* genus is one of the species known to contaminate food. In this case, *A. ovalis* stood out because it inhibited the growth of *A. brasiliensis*, suggesting a potential use as a natural antifungal preservative. However, more studies are recommended in this regard. Antibacterial activity cannot be directly compared with these authors because the methods used are different, however, earlier research as well as the present work showed that juneberry (*A. lamarckii*) presented antibacterial activity. In the present study the strongest inhibition was obtained against *Salmonella* spp.

The cytotoxic activity of *A. ovalis* was evaluated in non-tumor hepatic cells (PLP2). The extract did not exhibit the capacity to inhibit cell proliferation at the maximum concentration tested (400 µg/mL). Similar results were reported for *Carissa macrocarpa* (Eckl.) A. DC [34]. Thus, according to the obtained results, this species extract does not exhibit in vitro cytotoxicity in the tested hepatic model. To the authors knowledge there are no more studies about the cytotoxicity of this species; nevertheless, while these preliminary findings are promising, they are insufficient to stablish a broad safety profile, further toxicological studies, including in vivo assays are needed to stablish safe consumption parameters.

## Conclusions

*Amelanchier ovalis* is a wild fruit with a distinctive phytochemical profile characterized by significant levels of polyunsaturated fatty acids profile, tocopherol content, organic acids and total phenolic compounds. The fruits of the serviceberry are a source of sorbitol, a natural edulcorate, which further highlights its potential nutritional value. The results of the present study demonstrated that the edible portion of the fruit present significant antioxidant capacity in both chemical and biological in vitro assays, alongside inhibitory effects against selected bacterial and fungal strains. These findings suggest that *A. ovalis* could serve as a potential preservative ingredient, as well as a potential functional ingredient due to its natural bioactive compounds (*e.g*., tocopherols, essential fatty acids, among others).

Future research should evaluate the bioavailability of these bioactive compounds through simulated digestion models and in vivo studies to validate their physiological efficacy. Furthermore, investigating the specific compounds responsible for the antimicrobial activity could provide a preliminary basis for the development of natural preservatives in food or agricultural applications, possibly contributing to the objectives of the Sustainable Development Goals (SDGs), while, at the same time, recovering its traditional use as food and food ingredient as part of the Mediterranean diet and promoting sustainable local agricultural practices.

## Supplementary Information

Below is the link to the electronic supplementary material.

Supplementary File 1 (DOCX 60.0 KB)

## Data Availability

No datasets were generated or analysed during the current study.
