# Supplementary material for: Wild Fruits of Amelanchier ovalis Medik. (Rosaceae): A Valuable Neglected Resource of Nutrients and Bioactive Compounds
Source: Plant Foods Hum Nutr. 2026 Apr 10;81(2):44. doi: 10.1007/s11130-026-01497-x (PMC13068719; doi:10.1007/s11130-026-01497-x)
Supplement: Supplementary file 1 — (DOCX 60.0 KB) [file 11130_2026_1497_MOESM1_ESM.docx]

**SUPPLEMENTARY MATERIAL**

**Wild fruits of *Amelanchier ovalis* Medik. (Rosaceae): a valuable neglected resource of nutrients and bioactive compounds**

García-Herrera, P.^1,3^; Vega, E.N.^1^; Alonso-Esteban, JI.^1,3^; Tardío, J.^2^; Molina, A.K.^3^; Mandim, F. ^3^; Pires, Tânia C.S.P. ^3^; Barros, L.^3^; Pérez-Rodríguez, M.L. ^1^, Cámara, M.^1^, Fernández-Ruiz, V.^1^; Morales, P.^1,*^

^1^ Dpto. Nutrición y Ciencia de los Alimentos, Facultad de Farmacia, Universidad Complutense de Madrid (UCM), Plaza Ramón y Cajal s/n, 28040 Madrid, Spain.

^2^ Instituto Madrileño de Investigación y Desarrollo Rural, Agrario y Alimentario (IMIDRA), Finca El Encín. Apdo. 127, 28800 Alcalá de Henares, Madrid, Spain.

^3^ CIMO, LA SusTEC, Instituto Politécnico de Bragança, Campus de Santa Apolónia, 5300-253 Bragança, Portugal.

* Corresponding author: Patricia Morales (e-mail: [patricia.morales@farm.ucm.es](mailto:patricia.morales@farm.ucm.es))

1. **Material and methods**
   1. **Samples**

The fruits were collected in two wild populations of *Amelanchier ovalis* Medik. of Central Spain (see Table S1). The samples were harvested at the optimal maturing state, in July 2022, when the fruits were completely ripened, in accordance with permits Ref. PN-NC_022022 and Ref. ABSCH-IRCC-ES-262067-1 issued by the Spanish Ministry of Agriculture, Fisheries, and Food (MAPA). Samples from both origins were pooled and processed as a single batch. A portion of the fresh fruit mixture was reserved for immediate analysis of moisture, °Brix, pH, and titratable acidity. The remaining fruit was frozen and subsequent freeze-drying at –80 ºC and 0.029 mbar (Freezone; 4.5 L; LABCONCO, Fort Scott, KS, USA). Seeds were then removed to obtain a homogeneous analytical matrix corresponding to the dry edible portion (peel and pulp) of the fruit. The freeze-dried samples were stored at -20 °C (in dark and dry conditions) for subsequent analysis. Results obtained from freeze-dried samples were converted to fresh weight (fw) basis, considering the initial moisture content of the fruit samples, using the following formula: Value_fw_ = Value_dw_ X ((100 - % moisture)/ 100).

**Table S1.** Collection sites, coordinates and ecological conditions were *Amelanchier ovalis* fruits were sampled

| **Municipality (province)** | **Geographical coordinates** | **Ecological conditions** |
| --- | --- | --- |
| Valdemeca (Cuenca, Spain) | Latitude: 40° 14’ 47.9” N;  Length: 1° 45’ 39,6” W | Limestone soils, 1300 m |
| Bustarviejo (Madrid, Spain) | Latitude: 40° 50’ 54.5” N  Length: 3° 41’ 38.9” W | Siliceous soils, 1100 m |

- 1. **Chemical and Standard Solutions**

Internal standard (IS, tocol) was purchased in Matreya, Pleasant Gap, PA, USA. Reference standard mixture 37 (FAME) and standards of cyanidin-3-*O*-glucoside, ferulic acid and 6-hydroxy-2,5,7,8-tetramethylchroman-2-carboxylic acid (trolox) were purchased from Sigma-Aldrich, St. Louis, MO, USA. *n*-hexane, ethyl acetate and H_2_SO_4_ were purchased from Merck, Darmstadt, Germany. NaOH, Fast blue BB, Gallic acid, formic acid, methanol, KCl, CH_3_COONa, Folin ciocâlteu, Na_2_CO_3_, 2,2-difenil-1-picrilhidrazilo (DPPH), Dulbecco’s modified Eagle’s medium (DMEM), Phosphate Buffered Saline (PBS), 2,2′-azobis(2-methylpropionamidine) dihydrochloride (AAPH) were obtained from Sigma-Aldrich, St. Louis, MO, USA. *Enterobacter cloacae* (ATCC 49741), *Escherichia coli* (ATCC 25922), *Pseudomonas aeruginosa* (ATCC 9027), *Salmonella enterica* *subsp. enterica* serovar Enteriditis (ATCC 13076), *Yersinia enterocolitica* (ATCC 8610), *Bacillus cereus* (ATCC 11778), *Listeria monocytogenes* (ATCC 19111), *Staphylococcus aureus* (ATCC 25923), *Aspergillus fumigatus* (ATCC 204305) and *Aspergillus brasiliensis* (ATCC 16404) were acquired from Frilabo (Porto, Portugal)

- 1. **Physico-chemical** **analysis**
     1. *Moisture*

Moisture determination was carried out by loss of mass on drying according to AOAC method 984.25 [1]. Briefly, 2.5 g of the sample was weighted, and it was placed in dried capsules for 24 h in an oven at 100 ± 5 ºC until total water elimination. Values were expressed as g/100g fw. The determination was performed by triplicate (n=3).

- - 1. *pH*

pH determination was performed according to the AOAC 981.12 method [1] by potentiometric measure with a pH-meter MicropH – 2000 (Crison Instruments) Briefly, 2.5 g of the sample were mixed with 25 mL of water and allowed to rest for 10 min, after which the pH was measured. The determination was performed in triplicate (n=3).

- - 1. *Titratable acidity*

Titratable acidity (TA) was determined according to the AOAC 942.15 method [1]. For that, the sample previously prepared for pH measurement was neutralized with NaOH 0.1 N until a pH of 8.1 was reached. Results were expressed as mL of NaOH 0.1 N needed to neutralize the acids contained in 100 g of sample (fw). The analysis was performed in triplicate (n=3).

- - 1. *Brix degrees*

Brix degrees (°Bx) were measured with a digital refractometer (PR-1, Atago). A few drops from the supernatant from the sample previously prepared for the pH measurement (2.5 g of sample in 25 mL of water) were measured in the refractometer by triplicate. The determination was carried out by triplicate (n=3).

- 1. Chemical characterization
     1. *Fatty acids*

The fatty acids composition was evaluated using gas-liquid chromatography with flame ionization detection (YOUNG IN Chromass 6500 GC System, YL Instruments, Anyang, Korea), based on the methodology reported by Morales et al. [2], after fatty acid methyl esters (FAME) preparation by trans-esterification of the lipid fraction attained by Soxhlet extraction. The identification and quantification were determined by comparing the relative retention times of the FAME peaks of the samples with commercial standards (FAME). The results recorded were processed using CSW 4.0 Software (Informer Technologies, Inc., Solihull, UK) and were expressed as the relative percentage for each detected fatty acid compound. Results were expressed as relative percentages (%). The determination was carried out by triplicate (n=3).

- - 1. *Tocopherols*

Tocopherol composition according to the procedures described by Morales et al. [2]. A high-performance liquid chromatography system (HPLC, Knauer, Smartline system 1000, Berlin, Germany) coupled to a fluorescence detector (FP-2020; Jasco, Easton, PA, USA) programmed at 290 nm and 330 nm was used for the separation and quantification of the compounds (Table S2). Quantification was carried out based on the fluorescence signal response using the internal standard (tocol), whereas the identification of the compounds was performed using authentic standards. The results were expressed in mg per 100 g (fw). The determination was carried out by triplicate (n=3).

- - 1. *Soluble sugars*

The soluble sugars were extracted and analysed according to the methodology reported by Morales et al. [3]. The separation of the soluble sugars was performed by the HPLC coupled with a refraction index detector (Knauer Smartline 2300, Berlin, Germany). After separation, the free sugar compounds were identified, and quantified, by comparison with standards (Table S2), melezitose was used as an internal standard. Raw data were processed through the Clarity 2.4 software package (DataApex, Prague, Czech Republic), and the results were expressed in g per 100 g (fw). The determination was carried out by triplicate (n=3).

- - 1. *Organic acids*

According to the procedures described by Morales et al. [3], the organic acid composition was determined using ultra-high-performance liquid chromatography coupled with a diode array detector (UHPLC–DAD, Shimadzu 20A series UHPLC, Shimadzu Corporation, Kyoto, Japan). After the identification of the compounds, based on the comparison of the retention times and spectra of commercial standards, they were quantified by comparing peak areas recorded at 215 and 280 nm, as presented in Table S2. Results were processed using LabSolutions Multi LC-PDA software (Shimadzu Corporation, Kyoto, Japan) and were expressed in g per 100 g (fw). The determination was carried out by triplicate (n=3).

Table S2. Calibration curves used to quantify organic acids, soluble sugars and tocopherols.

| **Standard** | **Concentration range** | **Equation** | ***r*²** | **Limit of detection** | **Limit of quantification** |
| --- | --- | --- | --- | --- | --- |
| Oxalic acid  (mg/mL) | 1.2500 – 0.0098 | y = 8.0E+06 x + 331789 | 0.9912 | 0.1451 | 0.4398 |
| Quinic acid  (mg/mL) | 10 – 0.078125 | y = 692575 x + 11551 | 0.9983 | 0.5155 | 1.5622 |
| Malic acid  (mg/mL) | 10 – 0.078125 | y = 942562 x + 38506 | 0.9987 | 0.4431 | 1.3428 |
| Shikimic acid  (mg/mL) | 0.5 – 0.00390625 | y = 5.0E+07 x + 567119 | 0.9903 | 0.0610 | 0.1848 |
| Citric acid  (mg/mL) | 5 – 0.0390625 | y = 968367 x - 12295 | 0.9974 | 0.3169 | 0.9606 |
| Ascorbic acid  (mg/mL) | 0.25 – 0.015625 | y = 5.0E+07 x + 449262 | 0.9813 | 0.0502 | 0.1520 |
| Glucose  (mg/mL) | 24 – 0.3750 | y = 0.99618 x | 0.9998 | 0.1375 | 0.4167 |
| Fructose  (mg/mL) | 24 – 0.3750 | y = 0.85061 x | 0.9996 | 0.1847 | 0.560 |
| Sorbitol  (mg/mL) | 24 – 0.3750 | y = 1.24509 x | 0.9713 | 1.4689 | 4.4512 |
| Sucrose  (mg/mL) | 24 – 0.3750 | y = 0.93201 x | 0.9997 | 0.1552 | 0.4704 |
| Alpha tocopherol  (μg/mL) | 0.1250 – 8.0 | y = 0.44611 x | 0.9986 | 0.103 | 0.312 |
| Beta tocopherol  (μg/mL) | 0.1250 – 8.0 | y = 0.16221 x | 0.9994 | 0.070 | 0.212 |

- - 1. *Phenolic compounds*

For determining the total phenolic compound and the phenolic families, was used the QUENCHER (QUick, Easy, New, CHEap and Reproducible) methodology [4] which allows the quantification of both the soluble and insoluble compounds, since a small part of the homogenized samples (0.037 mm particle size) is put in direct contact with the reagents of each determination. The determination was carried out by triplicate (n=3).

*Total phenolic compounds*

The quantification of total phenolic compounds (TPC) was carried out using the Fast Blue BB assay, following the procedure described by Medina [5] and Palombini et al. [6], with slight adjustments. Briefly, 1.0 ± 0.5 mg of sample was mixed sequentially with 0.4 mL of 0.1% Fast Blue BB solution, 0.4 mL of 5% NaOH, and 4 mL of distilled water, homogenizing with a vortex after each addition. The mixture was then subjected to orbital shaking for 45 min, centrifuged at 6500 rpm for 10 min, and filtered. Absorbance was measured at 420 nm using a UV–vis spectrophotometer (Synergy HTX, Biotek). All analyses were performed in triplicate. A standard calibration curve was prepared with gallic acid (Table S3), and results were expressed as mg gallic acid equivalents (GAE) per 100 g (fw).

*Q-Hydroxybenzoic acids*

Hydroxybenzoic acids (HBA) were quantified following the procedure described by Bonoli et al. [7], with minor adjustments. Approximately 1.0 ± 0.5 mg of the sample was combined with 0.5 mL of distilled water and 4 mL of 3% formic acid, vortexed, and subsequently incubated on an orbital shaker. After 15 min, the mixture was centrifuged at 65,000 rpm for 5 min, filtered, and the absorbance was recorded at 280 nm using quartz cuvettes in a UV–vis spectrophotometer (Synergy HTX, Biotek). All assays were performed in triplicate. A calibration curve prepared with gallic acid was used (Table S3), and results were expressed as mg gallic acid equivalents (GAE) per 100 g (fw).

*Q-Hydroxycinnamic acids*

Hydroxycinnamic acids (HCA) were analysed following the method of Bonoli et al. [7]. In summary, 1.0 ± 0.5 mg of sample was combined with 0.5 mL of distilled water and 4 mL of methanol, then vortexed and agitated in an orbital shaker for 15 min. The mixture was subsequently centrifuged at 6500 rpm for 5 min, filtered, and its absorbance recorded at 320 nm using a UV–vis spectrophotometer (Synergy HTX, Biotek). All analyses were performed in triplicate. A standard curve prepared with ferulic acid served for quantification (Table S3), and the results were expressed as mg ferulic acid equivalents (FAE) per 100 g (fw).

*Q-Flavonols*

Flavonol content (FLAV) was assessed following the procedure described by Bonoli et al. [7]. In short, 1.0 ± 0.5 mg of sample was mixed with 0.5 mL of distilled water and 4 mL of methanol, vortexed, and shaken in an orbital shaker for 15 min. The mixture was then centrifuged at 6500 rpm for 15 min, filtered, and its absorbance was recorded at 370 nm using a UV–vis spectrophotometer (Synergy HTX, Biotek). A calibration curve prepared with quercetin was used (Table S3), and results were expressed as mg quercetin equivalents (QE) per 100 g (fw).

*Q-Total anthocyanin content*

Total anthocyanin content (TAC) was determined using the pH-differential method described by Vega et al. [8]. Briefly, 10 ± 0.1 mg of sample was diluted in 10 mL of either KCl buffer (pH 1.0) or sodium acetate buffer (pH 4.5), vortexed, and shaken on an orbital shaker for 15 min. The mixtures were then centrifuged at 7000 rpm for 5 min, filtered, and their absorbance measured at 510 and 700 nm with a UV–vis spectrophotometer (Synergy HTX, Biotek). A calibration curve was generated using cyanidin-3-*O*-glucoside (Table S3), and the results were expressed as mg cya-3-glu per 100 g (fw).

Table S3. Calibration curves used to quantify TPC, phenolic families and TAC.

| **Standard** | **Concentration range (µg/mL)** | **Equation** | ***r*²** | **Limit of detection**  **(mg/mL)** | **Limit of quantification**  **(mg/mL)** |
| --- | --- | --- | --- | --- | --- |
| TPC  (mg GAE/100 g, fw) | 0.625 – 160 | y=0.0074x+0.0194 | 0.999 | 1.6093 | 4.8765 |
| HBA  (mg GAE/100 g, fw) | 3.125 – 400 | y=0.0467x – 0.0058 | 0.9997 | 0.0308 | 0.0935 |
| HCA  (mg FAE/100 g, fw) | 3.125 – 200 | y=0.0867x - 0.0462 | 0.9998 | 0.1582 | 0.4793 |
| FLAV  (mg quercetin/100 g, fw) | 3.125 – 250 | y=0.0262x - 0.0147 | 0.9984 | 1.6552 | 5.0158 |
| TAC  (mg cya-3-glu/100 g, fw) | 0.156 – 25 | Y=0.036x - 0.0009 | 0.9994 | 0.3039 | 0.9208 |

- 1. **Antioxidant Capacity and Biological Activities**
     1. *Antioxidant properties*

Overall antioxidant capacity was also performed using the QUENCHER methodology, applied to Folin-Ciocâlteu, DPPH, and FRAP methods, avoiding solvent extraction or a hydrolysis step, given that the reagents act directly on the sample. Moreover, Oxidative Haemolysis Inhibition Assay (OxHLIA) was performed to study antioxidant properties in cellular models. The determination was carried out by triplicate (n=3).

*Q-Folin-Ciocâlteu Assay*

This method, which uses a reagent containing phosphomolybdic/phosphotungstic acid complexes, has been proposed as an antioxidant capacity analysis method rather than for the quantification of phenolic compounds [9] since not only phenolics, but also other reducing compounds such as ascorbic acid, may react with molybdenum, forming blue complexes that can be detected spectrophotometrically. Therefore, the methodology described by Slinkard and Singleton [10] was adapted, weighing 1 mg of dried sample and adding 0.8 mL of distilled water and 0.2 mL of Folin-Ciocâlteu reagent which was mixed by vortex, and after 5 min of reaction, 4 mL of Na_2_CO_3_ 0.7 M and 5 mL of distilled water were added, stirred by vortex and placed in an orbital shaker for 45 min, finally, the absorbance was measured at 750 nm against a blank. All measurements were performed in triplicate. A calibration curve of gallic acid was obtained (Table S4), thus, results were expressed as mg of GAE/ 100 g (fw).

*Q-DPPH Assay*

The antioxidant capacity by the Q-DPPH method was determined following the methodology proposed by Del Pino-Garcia et al. [4]. Reaction mechanism is based on an electron transfer reaction and in this method, the purple chromogen radical 2,2- diphenyl-1-picrylhydrazyl (DPPH^•^) is reduced by antioxidant/reducing compounds to the corresponding pale-yellow hydrazine. A 0.1 mM DPPH solution was diluted with ethanol/water (50:50, v/v) until reaching an absorbance at 517 nm between 0.75 and 0.80. Then, the sample was weighed (2 ± 0.1 mg) and 10 mL of the DPPH dilution were added, the sample was stirred in a vortex (Velp Scientifica, Usmate, Italy) and kept in an orbital shaker for 1 h. Then it was centrifuged for 5 min at 7,000 rpm, filtered and measured at 517 nm in a Synergy HTX multi-mode reader spectrum using microplates. Trolox was used as standard to perform a calibration curve (Table S4), and results were expressed as mg TE/ 100 g (fw).

*Q-FRAP Assay*

The methodology described by Benzie & Strain [11] was used for the determination of the Fe (III) reduction capacity by Q-FRAP analysis, with modifications from Del Pino-Garcia et al. [4]. To 2 mg of sample were added 40 mL of FRAP reagent, and it was incubated at 37 ºC for 30 min with continuous stirring. After centrifugation at 7000 rpm for 5 min and filtration, the absorbance was measured at 595 nm in a Synergy HTX multi-mode reader spectrum. Trolox was used as a standard to obtain a calibration curve (Table S4), and the results were expressed as mg TE/ 100 g (fw).

**Table S4**. Calibration curves used to quantify the *in vitro* Total Antioxidant Capacity

| **Standard** | **Concentration range** (µg/mL) | **Equation** | ***r*²** | **Limit of detection**  **(mg/mL)** | **Limit of quantification**  **(mg/mL)** |
| --- | --- | --- | --- | --- | --- |
| Q-Folin-Ciocâlteu  (mg GAE/100 g fw) | 50 – 400 | y=0.0879x+0.0254 | 0.9969 | **0.20571** | **0.6234** |
| Q-DPPH  (mg TE/100 g fw) | 12.5 – 200 | y=12.261x – 0.099 | 0.9998 | **0.08379** | **0.2539** |
| Q-FRAP  (mg TE/100 g fw) | 2.125 – 250 | y = 0.1296x + 0.0004 | 0.999 | **0.1840** | **0.5575** |

*Oxidative Haemolysis Inhibition Assay (OxHLIA)*

Another way of measuring the antioxidant activity was by determining their anti-haemolytic activity, according to Vega et al. [8] (2023). An erythrocyte solution (2.8%, v/v; 200 µL) prepared in phosphate-buffered saline (PBS, pH 7.4) was mixed with 400 µL of either: different concentrations of the fruit extract solution (4.69 – 600 µg/mL in PBS); PBS as negative control; distilled water for baseline; or different concentrations of Trolox (7.81 – 250 µg/mL in PBS), which served as the positive control. After pre-incubation at 37 °C for 10 min with shaking, 200 μL of 2,2'-azobis(2-methylpropionamidine) dihydrochloride (AAPH, 160 mM in PBS) was added, and the optical density was measured over time until complete haemolysis at 690 nm in an ELx800 microplate reader (Bio-Tek Instruments, Winooski, VT, USA). Results were expressed as IC_50_ values (µg/mL) representing the concentration required to delay haemolysis by 60 or 120 min (∆t 60 and ∆t 120). The determination was carried out by triplicate (n=3).

- - 1. *Antibacterial activity*

The antibacterial activity was evaluated against a panel of foodborne bacteria. Five Gram-negative strains were included: *Enterobacter cloacae*, *Escherichia coli*, *Pseudomonas aeruginosa*, *Salmonella enterica* *subsp. enterica* serovar Enteriditis, and *Yersinia enterocolitica*. In addition, three Gram-positive strains were tested: *Bacillus cereus*, *Listeria monocytogenes*, and *Staphylococcus aureus*. Before analysis, bacterial cultures were grown in fresh medium at 37 °C for 24 h to ensure exponential growth. Minimum inhibitory concentration (MIC) values were determined using the colorimetric method of Pires et al. (2018). Stock solutions (20 mg/mL) were prepared by dissolving samples in 5% (v/v) DMSO and 95% sterile distilled water. For the assay, 100 μL of sample solution was added to the first well of a 96-well microplate, along with 90 μL of Tryptic Soy Broth (TSB). Each subsequent well contained 90 μL of TSB, and serial dilutions were performed to obtain final concentrations ranging from 10 to 0.03125 mg/mL. Finally, 10 μL of bacterial inoculum (standardized to 1.5 × 10^6^ CFU/mL, yielding 1.5 × 10^5^ CFU per well) was added. All assays were performed in duplicate. Negative controls consisted of TSB alone and TSB with extract, while positive controls included TSB with inoculum, as well as TSB with bacteria and antibiotics. Ampicillin and streptomycin were tested against all bacteria, with methicillin additionally used for *S. aureus*. Microplates were incubated at 37 °C for 24 h. MIC values were assessed after adding 40 μL of INT (0.2 mg/mL) followed by incubation at 37 °C for 30 min. MIC was defined as the lowest concentration preventing visible growth, indicated by the absence of color change (yellow to pink). Minimum bactericidal concentration (MBC) was determined by plating 10 μL from wells showing no color change onto Blood Agar (7% sheep blood) and incubating at 37 °C for 24 h. The MBC corresponded to the lowest concentration at which no bacterial growth was observed, indicating complete bactericidal activity. The determination was carried out by triplicate (n=3).

- - 1. *Antifungal activity*

Antifungal activity was evaluated following the methodology described by Vega et al. [8]. Two *Aspergillus* species, *Aspergillus fumigatus* and *Aspergillus brasiliensis,* common contaminants of food-related plant materials, were used in the assays. Strains were maintained on malt agar at 4 °C. Before to testing, cultures were transferred to fresh medium and incubated at 25 °C for 72 h. For spore preparation, conidia were harvested from agar plates using sterile 0.85% saline solution supplemented with 0.1% Tween 80 (v/v). The suspension was adjusted to approximately 1.0 × 10^5^ spores in a final volume of 100 μL per well. Sample stock solutions (20 mg/mL) were prepared by dissolving extracts in 5% (v/v) DMSO and 95% sterile distilled water. In the assay, 100 μL of stock solution was added to the first well of a 96-well microplate containing 90 μL of Malt Extract Broth (MEB), in duplicate. Each subsequent well received 90 μL of MEB, and serial dilutions were performed to achieve concentrations ranging from 10 to 0.03125 mg/mL. Minimum inhibitory concentrations (MICs) were determined using the microdilution method in 96-well plates. The MIC was defined as the lowest concentration without visible fungal growth, verified under a binocular microscope. Minimum fungicidal concentration (MFC) was assessed by transferring 2 μL from wells showing no visible growth into fresh microplates with 100 μL of MEB per well, followed by incubation at 26 °C for 72 h. The MFC was the lowest concentration at which no fungal growth occurred, indicating 99.5% reduction of the initial inoculum. Ketoconazole (Frilabo, Porto, Portugal) served as the positive control. The determination was carried out by triplicate (n=3).

- - 1. *Antiproliferative Activity in Non-Tumour Cells*

The antiproliferative activity was determined using a primary culture established from porcine liver cells (PLP2) [12]. The cells’ proliferation was monitored using a phase contrast microscope. When cells reached 80 to 90% confluence, were trypsinized and transferred to 96 well microplates (density of 1.0 × 10^4^ cells/well) with the culture medium Dulbecco’s modified Eagle’s medium (DMEM) supplemented with FBS (10%), penicillin (100 U/mL), and streptomycin (100 µg/mL). To determine the antiproliferative activity, the cells were incubated with different concentrations of the extract (between 400 and 6.25 µg/mL). The cells’ proliferation was quantified by the sulphorodamine B colorimetric assay. The results were expressed as GI_50_ values, which corresponded to the sample concentration (µg/mL) responsible for inhibiting cell proliferation in 50%. Ellipticine was used as a positive control. The cytotoxicity was assessed after the colorants preparation (t0) and after 12 weeks of storage at room and refrigerated temperatures, to guarantee their safety for food application. The determination was carried out by triplicate (n=3).

- 1. Statistical Analysis

All analyses were performed in triplicate (n=3), and results are expressed as mean values ± standard deviation. Calibration curves equations and r^2^ values were determined using Microsoft® Excel 2025. To calculate IC_50_ values for OxHLIA (µg/mL) at Δt of 60 and 120 min, extract concentrations were correlated against Δt values (min), which derived by subtracting the half-haemolysis time (Ht_50_) of the negative control from the Ht_50_ of each extract concentration, both of which were determined graphically using GraphPad Prism 8 (San Diego, CA, USA). Moreover, Spearman’s correlation was conducted using IBM SPSS version 29.

**References**

1. AOAC (2005) Official Methods of Analysis, 18th ed. AOAC International, Maryland, USA

2. Morales P, Ferreira ICFR, Carvalho AM, et al (2013) Wild edible fruits as a potential source of phytochemicals with capacity to inhibit lipid peroxidation. European Journal of Lipid Science and Technology 115:176–185. https://doi.org/10.1002/ejlt.201200162

3. Morales P, Barros L, Ramírez-Moreno E, et al (2015) Xoconostle fruit (*Opuntia matudae* Scheinvar cv. Rosa) by-products as potential functional ingredients. Food Chem 185:289–297. https://doi.org/10.1016/j.foodchem.2015.04.012

4. Del Pino-García R, García-Lomillo J, Rivero-Pérez MD, et al (2015) Adaptation and Validation of QUick, Easy, New, CHEap, and Reproducible (QUENCHER) Antioxidant Capacity Assays in Model Products Obtained from Residual Wine Pomace. J Agric Food Chem 63:6922–6931. https://doi.org/10.1021/acs.jafc.5b01644

5. Medina MB (2011) Determination of the total phenolics in juices and superfruits by a novel chemical method. J Funct Foods 3:79–87. https://doi.org/10.1016/j.jff.2011.02.007

6. Palombini S V., Claus T, Maruyama SA, et al (2016) Optimization of a New Methodology for Determination of Total Phenolic Content in Rice Employing Fast Blue BB and QUENCHER Procedure. J Braz Chem Soc 27:1188–1194. https://doi.org/10.5935/0103-5053.20160013

7. Bonoli M, Verardo V, Marconi E, Caboni MF (2004) Antioxidant Phenols in Barley (*Hordeum vulgare* L.) Flour:  Comparative Spectrophotometric Study among Extraction Methods of Free and Bound Phenolic Compounds. J Agric Food Chem 52:5195–5200. https://doi.org/10.1021/jf040075c

8. Vega EN, García-Herrera P, Ciudad-Mulero M, et al (2023) Wild sweet cherry, strawberry and bilberry as underestimated sources of natural colorants and bioactive compounds with functional properties. Food Chem 414:135669. https://doi.org/10.1016/j.foodchem.2023.135669

9. López-Froilán R, Hernández-Ledesma B, Cámara M, Pérez-Rodríguez ML (2018) Evaluation of the Antioxidant Potential of Mixed Fruit-Based Beverages: a New Insight on the Folin-Ciocalteu Method. Food Anal Methods 11:2897–2906. https://doi.org/10.1007/s12161-018-1259-1

10. Slinkard K, Singleton VL (1977) Total Phenol Analysis: Automation and Comparison with Manual Methods. Am J Enol Vitic 28:49–55. https://doi.org/10.5344/ajev.1977.28.1.49

11. Benzie IFF, Strain JJ (1996) The Ferric Reducing Ability of Plasma (FRAP) as a Measure of “Antioxidant Power”: The FRAP Assay. Anal Biochem 239:70–76. https://doi.org/10.1006/abio.1996.0292

12. Mandim F, Graça VC, Calhelha RC, et al (2019) Synthesis, Photochemical and In Vitro Cytotoxic Evaluation of New Iodinated Aminosquaraines as Potential Sensitizers for Photodynamic Therapy. Molecules 24:863. https://doi.org/10.3390/molecules24050863
